# Construction of a Humanized Artificial VHH Library Reproducing Structural Features of Camelid VHHs for Therapeutics

**DOI:** 10.3390/antib11010010

**Published:** 2022-01-30

**Authors:** Taihei Murakami, Shigefumi Kumachi, Yasuhiro Matsunaga, Miwa Sato, Kanako Wakabayashi-Nakao, Hidekazu Masaki, Ryo Yonehara, Maiko Motohashi, Naoto Nemoto, Masayuki Tsuchiya

**Affiliations:** 1 Epsilon Molecular Engineering, Inc., Saitama 338-8570, Japan; murakami_t@epsilon-mol.co.jp (T.M.); kumachi@epsilon-mol.co.jp (S.K.); nakao@epsilon-mol.co.jp (K.W.-N.); masaki@epsilon-mol.co.jp (H.M.); yonehara@epsilon-mol.co.jp (R.Y.); motohashi@epsilon-mol.co.jp (M.M.); nemoto@epsilon-mol.co.jp (N.N.); 2 Graduate School of Science and Engineering, Saitama University, Saitama 338-8570, Japan; ymatsunaga@mail.saitama-u.ac.jp; 3 Mitsui Knowledge Industry Co., Ltd., Tokyo 164-0003, Japan; sato-miwa@mki.co.jp

**Keywords:** variable domain of heavy chain antibodies, synthetic library, cDNA display, therapeutic antibodies, high throughput screening

## Abstract

A variable domain of heavy chain antibody (VHH) has different binding properties than conventional antibodies. Conventional antibodies prefer binding to the convex portion of the antigen, whereas VHHs prefer epitopes, such as crevices and clefts on the antigen. Therefore, developing candidates with the binding characteristics of camelid VHHs is important. Thus, To this end, a synthetic VHH library that reproduces the structural properties of camelid VHHs was constructed. First, the characteristics of VHHs were classified according to the paratope formation based on crystal structure analyses of the complex structures of VHHs and antigens. Then, we classified 330 complementarity-determining region 3 (CDR3) structures of VHHs from the Protein Data Bank (PDB) into three loop structures: *Upright*, *Half-Roll*, and *Roll*. Moreover, these structures depended on the number of amino acid residues within CDR3. Furthermore, in the *Upright* loops, several amino acid residues in the FR2 are involved in the paratope formation, along with CDR3, suggesting that the FR2 design in the synthetic library is important. A humanized synthetic VHH library, comprising two sub-libraries, *Upright* and *Roll*, was constructed and named *PharmaLogical*. A validation study confirmed that our *PharmaLogical* library reproduces VHHs with the characteristics of the paratope formation of the camelid VHHs, and shows good performance in VHH screening.

## 1. Introduction

In 1993, it was reported that camelids produced unconventional heavy-chain-only antibodies that bind to antigens solely by the variable domain of their heavy chain. The *variable domain of heavy chain of heavy chain antibody* (VHH) was expected to be the next generation of therapeutic antibodies [1]. In those days, however, fierce competition in the development of conventional human or humanized antibodies had only begun, and thus, a few pharmaceutical companies were seriously looking at camelid VHHs. Among them, *Ablynx* (a subsidiary of Sanofi since 2018) generated several VHH clinical candidates, including *caplacizumab*. Therefore, many were surprised when *caplacizumab*, the world’s first VHH drug, was approved as a treatment for thrombotic thrombocytopenia by the European Medicines Agency in 2018, and by the Food and Drug Administration (FDA) in 2019 [2,3]; after that, many pharmaceutical companies turned their attention to VHH research and development. In March 2021, in Japan, a second VHH drug, *ozoralizumab*, was submitted to the Pharmaceuticals and Medical Devices Agency for approval against rheumatoid arthritis.

Although several scaffolds, such as *affibodies* [4] and *DARPins* [5], have been studied for a long time as next-generation biologics, only low-molecular-weight antibody single-chain fragments (scFv) were eventually applied to bispecific T cell engager (*BiTE*^®^) [6] and chimeric antigen receptor T cells (CAR-T) [7] modalities in cancer therapy. However, they have not shown enough impact to cause a paradigm shift in the antibody drug market.

It has been revealed that VHH is a particularly successful scaffold as a drug-discovery molecule [8]. High affinity and specificity, comparable to conventional antibodies, low molecular weight, and various physicochemical properties, such as structure and thermal stabilities, are suitable for developing biologics, intrabody application, and efficient production in a microbial host.

The most attractive modality is bispecific antibodies that bind to different targets simultaneously. Future biopharmaceuticals require a multi-paratopic characteristic that binds to multiple sites on the same target. However, it is difficult to construct multi-paratopic antibodies due to their molecular size. Alternatively, VHHs are small-sized (~14 kDa) with high stability (T_m_ up to 90 °C) [9], are easy to design, and construct multi-paratopic molecules which simultaneously bind to different epitopes on the same target that will significantly enhance affinity and specificity and shows multi-functional activities [10]. Indeed, most VHHs currently under clinical development have a multi-paratopic structure [11].

Although camelid VHHs were suspected to be immunogenic in humans, the humanization of those with high homology (about 80%) with human VH family 3 is thought to not be troublesome [12]. Their short plasma half-life (about 40 min, due to renal excretion) is of concern, but technology that prolongs their plasma half-life has already been applied in clinical practice [13].

VHHs are conventionally obtained by immunizing camelids such as llamas and alpacas with the target protein, but this method is time-consuming, the outcome is often unpredictable depending on the target, and animal use is increasingly being restricted, especially in the European Union [14]. To obtain VHHs faster and more efficiently, VHH libraries have been established and screened for binders by phage and yeast displays [15,16,17,18,19]. However, the diversity of cell-based systems is restricted by limited transformation efficiency (typically up to 10^8–9^). Conversely, in vitro cell-free approaches, such as cDNA display [20], mRNA display [21,22], and ribosome display [23], have been developed with high diversity (10^13–15^) and are used for VHH screening. The cDNA display method was evaluated as the most robust screening performance [24]. A synthetic VHH library, in which three complementarity-determining regions (CDRs) were randomized, was constructed, and several VHHs against survivin were obtained from the VHH library using cDNA display screening, indicating that the cDNA display method could be used to functionally screen synthetic VHHs [25]. Additionally, Hidayah et al. also reported the construction of a semisynthetic human VHH library [26,27]. In that study, CDRs were randomized based on the human antibody VH3-23 DP-47. These artificial synthetic libraries worked well to obtain binders for the antigens. However, the performance of that library is still unclear, and there is no certainty that the library can reproduce the characteristics of camelid VHHs.

Here, we report a humanized artificial synthetic VHH library that retains the structural features contributing to the paratope formation on the camelid VHHs. We analyzed 330 VHH crystal structures registered in the Protein Data Bank (PDB), obtained the features of each paratope on camelid VHH, and then classified the CDR3 structures into three types; *Upright*, *Roll*, and *Half-Roll*. Based on these analyses, four sub-libraries were constructed and designated, according to the structure type, as *Upright 6*, *Upright 12*, *Roll 12*, and *Roll 15*. The numbers in the name indicate the number of amino acid residues in the CDR3s. To minimize heterogeneity in the development of biologics, we reduced the frequency of the appearance of amino acids susceptible to any modification and the amino acids that may affect the structure, such as cysteine, methionine, and proline. For pharmaceutical purposes, the FRs were humanized according to the human/humanized sequences from, for example, DP-47. This library has the camelid structural features of paratope, humanized FRs, and optimized amino acid sequences in CDR3 to reduce a possible heterogeneity and was named by the *PharmaLogical* library.

Next, to validate the library performance, we conducted VHH screenings against liver cancer antigen glypican-3 (GPC3) [28] and breast cancer antigen HER2 [29] using cDNA display technology. Consequently, various VHHs with affinities from 10 pM to 100 nM were rapidly isolated for both antigens. Furthermore, molecular dynamics simulations of isolated VHHs from the sub-libraries confirmed that the CDR3s of the library certainly take the expected types (i.e., *Upright* and *Roll*) as their stable structures. These data indicated that our synthetic library reproduces the structural properties of the camelid VHHs. Therefore, our *PharmaLogical* library is useful for drug discovery to obtain VHHs with the original camelid antigen-binding properties.

## 2. Materials and Methods

### 2.1. Creation of Structural Classification Data

The structural information on antibodies was obtained from the SAbDab database (http://opig.stats.ox.ac.uk/webapps/newsabdab/sabdab/ (accessed on 20 June 2021)), which contains all antibody structures available in the PDB [30]. We extracted the VHH data and human VH data from this database according to the following criteria:

1. Chain IDs for heavy chains included, but light chains IDs not included;
2. Tertiary structures determined using X-ray crystallography with a resolution better than 2.8 Å;
3. Exclude structures in a complex with a hapten-antigen.

Criteria for human VH data:

1. Chain IDs for both heavy and light chains;
2. Tertiary structures determined using X-ray crystallography with a resolution better than 2.8 Å;
3. Heavy and light antibody chains were both of the species homo sapiens;
4. Exclude structures in a complex with a hapten-antigen.

The amino acid sequences of each chain were derived from the SEQRES records in the PDB files. The residue numbers were renumbered automatically by the Kabat numbering scheme, using the ANARCI tool [31] (http://opig.stats.ox.ac.uk/webapps/newsabdab/sabpred/anarci/ (accessed on 31 May 2021)). The residue number was used to identify the variable domain region and the CDR. Clustering of variable domain region, based on these amino acid sequences, was conducted using CD-HIT (http://weizhong-lab.ucsd.edu/cd-hit/ (version 4.8.1, accessed on 31 May 2021)) to eliminate redundancies by removing perfect matches for which the % id was 100% [32]. Data curation resulted in a VHH dataset of 330 and a human VH dataset of 926.

### 2.2. VHH and Human VH Datasets

The structure of CDR3 was classified into three groups (*Upright*, *Roll*, and *Half-Roll*) according to the structural feature using the following index based on Shirai [33] and Gray [34] (the classification does not apply to short CDR3s less than five amino acids. For those short CDR3s, the structures were visually classified from the X-ray structures):

1. Distance between Cα atoms: distance between the H46 residue and the (n −5)th residue, with H102 (endpoint of CDR3) as the (n)th residue;
2. Pseudo-dihedral angle (θbase): dihedral angle consisting of the four Cα atoms of the (n +1)th, (n)th, (n −1)th, and (n −2)th residues, with H102 (the endpoint of CDR3) as the (n)th residue.

### 2.3. Design of PharmaLogical Library

First, it was necessary to set the boundary between CDRs and FRs. We used the Kabat numbering rule [35] and structural classifications from Chothia [36]. Consequently, they were designated as FR1 (H1–H25), CDR1 (H26–H35), FR2 (H36–H49), CDR2 (H50–H65), FR3 (H66–H94), CDR3 (H95–H102), and FR4 (H103–H113).

Humanized FRs for each *Upright* and *Roll* type are designed based on the human framework sequence IGHV3-23 * 01 (DP-47), and VHHs on the market or under clinical development. We carefully determined the Vernier zone, which is the framework region interacting with the CDRs, based on the structural data of the VHHs. Additionally, the amino acid sequences of the FRs, especially FR2, were specialized for each *Upright* and *Roll* type.

Four sub-libraries were designed based on the features of the CDR3 structures, and named *Upright 6*, *Upright 12*, *Roll 12*, and *Roll 15*. CDR2, with 17 amino acid residues, prefers to form the *Roll* type, whereas CDR2, with 16 amino acid residues, prefers to form the *Upright* type. For CDR1, 150 varieties of amino acid sequences were selected; for CDR2, 69 and 71 varieties of sequences were selected in the *Roll* and *Upright* libraries, respectively. Alternatively, CDR3s were randomly designed with 17 amino acid residues, excluding methionine, proline, and cysteine. Finally, the CDR1s, CDR2s, and CDR3s were combined with humanized FRs.

### 2.4. Library Construction

First, we synthesized a DNA fragment encoding the FR1 including the 5′ UTR, the FR3 including a restriction enzyme *BtgZI* site at the 3′ terminus, and a DNA fragment encoding the FR4 including a His tag sequence and the 3′ UTR (Eurofins Genomics Inc., Eurofins Scientific SE, Luxembourg, Luxembourg). Next, 150 oligonucleotides, including CDR1, FR2, and a restriction enzyme *BsmBI* site at the 3′ terminus, were synthesized. In this construct, two types of oligonucleotides were prepared for each *Upright* and *Roll* types. For the *Upright* type, 71 oligonucleotides, including the CDR2, the FR2, and a restriction enzyme *BsmBI* site at the 5′ terminus, were synthesized, and for the *Roll* type, 69 oligonucleotides, including CDR2, FR2, and a restriction enzyme *BsmBI* site at the 5′ terminus, were synthesized (Sigma-Aldrich, Merck KGaA, Darmstadt, Germany). One hundred fifty oligonucleotides for the CDR1, 71 for the CDR2, and 69 for the CDR2 were mixed. Finally, oligonucleotides, including a randomized CDR3 region and a restriction enzyme *BsmBI* site at the 5′ terminus, were synthesized (Ella Biotech, Fürstenfeldbruck, Germany). The randomized regions included amino acid residues of lengths of 6, 12, or 15, and were synthesized using 17 types of trimer phosphoramidites (excluding cysteine, methionine and proline). Notably, these 17 types of trimer phosphoramidites were equally mixed.

Overlapping extension polymerase chain reactions (PCRs) were conducted with the mixed oligonucleotides (FR1 and CDR1), mixed oligonucleotides 2 (CDR2 and FR3), and mixed oligonucleotides 3 (CDR3 and FR4), 100 pmol per mixture. The extension PCRs synthesized strands of FR1-CDR1, CDR2-FR3, and CDR3-FR4. The FR1-CDR1 and the CDR2-FR3 were purified with *AMPure XP* (Beckman Coulter, Inc., Brea, CA, USA) and digested with a restriction enzyme *BsmBI* (New England Biolabs, Ipswich, MA, USA). After purification with *AMPure XP*, the FR1-CDR1 and the CDR2-FR3 fragments were ligated together with T4 DNA ligase (Takara Bio Inc., Shiga, Japan) at 16 °C overnight. The ligated products were purified by an 8 M urea denaturing polyacrylamide gel electrophoresis (PAGE) and amplified by PCR using the outer primers. The amplified DNA fragments of FR1-CDR1-FR2-CDR2-FR3 were purified. The purified products were digested with a restriction enzyme *BtgZI* (New England Biolabs), and DNA fragments of CDR3-FR4 were digested with a restriction enzyme *BsmBI* after purification with *AMPure XP*. The DNA fragments of FR1-CDR1-FR2-CDR2-FR3 and CDR3-FR4 were ligated together with T4 DNA ligase at 16 °C overnight. The ligated products were purified by an 8 M urea denaturing PAGE. The DNA library from our *PharmaLogical* library is composed of 5′ UTR (T7 promoter, Shine–Dalgarno sequence), VHH genes, the His-tag region, and 3′ UTR linker hybridization region.

### 2.5. Obtaining VHHs against Target Molecules

We built a high-throughput screening platform called “*The Month*” and conducted a verification study using that platform. The combination of the cDNA display method and the *Corynebacterium glutamicum* secretion expression system enabled the rapid isolation of many target-binding VHHs.

#### 2.5.1. In Vitro Selection Using cDNA Display

Each sub-library, according to DNA form *Upright 6*, *Upright 12*, *Roll 12*, and *Roll 15,* was transcribed by T7 RNA polymerase in a *RiboMAX Express Large Scale RNA Production System* (Promega Corp., Madison, WI, USA). The RNA products were purified with *RNAClean XP* (Beckman Coulter, Brea, CA, USA), and then hybridized with a puromycin linker using *cnvK*. The *cnvK* puromycin linkers were individually photo-cross-linked with the 3′-terminal region of the mRNA by ultraviolet light at 365 nm for 30 s.

The photo-cross-linked products were mixed and translated in vitro using *PUREflex1.0* (GeneFrontier Corporation, Chiba, Japan) at 30 °C for 30 min. KCl and MgCl_2_ were added to the reaction mixture at a final concentration of 800 and 80 mM, respectively, and incubated at 37 °C for 60 min, and then ethylenediaminetetraacetic acid (EDTA) was added to the reaction mixture at a final concentration of 100 mM, and the mixture was further incubated at 4 °C for 15 min. A 2× binding buffer (20 mM Tris-HCl pH 7.5, 2 M NaCl, 25 mM EDTA, 0.1% Tween20) was added to the translation reaction mixture. The reaction mixture was incubated with streptavidin-coated magnetic beads (SA beads, *Dynabeads^TM^, MyOne^TM^ Streptavidin C1*; Thermo Fisher Scientific, Inc., Waltham, MA, USA) at 25 °C for 60 min to immobilize on SA beads. After washing with 1× binding buffer three times, the immobilized library was reverse-transcribed by *GeneAce Reverse Transcriptase* (Nippon Gene, 200 U/μL *GeneAce Reverse Transcriptase* in 200 μL buffer) at 42 °C for 30 min. After washing twice with a His-tag wash buffer (20 mM sodium phosphate pH 7.4, 0.5 M NaCl, 5 mM imidazole, 0.05% Tween20), the His-tag wash buffer with 5 U/μL RNase T1 (Thermo Fisher) was mixed with the SA beads, and the mixture was incubated at 37 °C for 15 min to release the cDNA display molecules from the SA beads. The supernatant containing the cDNA display was captured with Ni-NTA magnetic beads (*His Mag Sepharose Ni^®^*, Cytiva, Marlborough, MA, USA) at 25 °C for 60 min. The Ni-NTA magnetic beads were then collected and washed twice with a His-tag wash buffer. The cDNA display molecules bound to the beads were eluted by incubation at 25 °C for 10 min in a His-tag elution buffer (20 mM sodium phosphate pH 7.4, 0.5 M NaCl, 250 mM imidazole, 0.05% Tween20) at 25 °C for 15 min.

At the first round of selection, cDNA display libraries were mixed with 100 pmol biotinylated HER2 or 100 pmol biotinylated GPC3, and incubated at 4 °C for 30 min. Then, 240 μL SA beads was added to the reaction mixtures and incubated at 4 °C for 30 min to immobilize the cDNA display molecules bound to the biotinylated target proteins. The supernatant was removed, and the beads were washed three times with PBS-T. The cDNA display molecules remaining on the beads were eluted and purified by *AMPure XP*. The purified products were subjected to PCR amplification. The amplified DNAs were purified and used for the next round of selection. For the second and third rounds of selection, a smaller amount of mRNA linker (12.5 pmol and 6.25 pmol, respectively), and a smaller amount of biotinylated target proteins (10 pmol HER2 and GPC3) were used.

#### 2.5.2. Preparation of Selected VHHs

After selection, cDNA fragments from the selected clones were PCR-amplified using specific primers, including restriction enzymes *BamHI* and *SfiI* restriction enzyme sites, and transferred into an expression vector for *Corynebacterium glutamicum*. Approximately 200 transformants were picked up and these were cultured in a protein-expression medium at 25 °C for 72 h. The culture supernatants were centrifuged and filtered through a 0. 22-µm membrane filter.

#### 2.5.3. Primary Binding Assay by Biolayer Interferometry Analysis

VHHs with binding activity against the target molecules were determined by using a biolayer interferometry (BLI), Octet^®^ RED384 (Sartorius AG, Göttingen, Germany). A solution of 70 μL was added to a 384-well black plate (Sartorius AG), and the measurements were conducted as described below. The loading baseline was measured in kinetic buffer for 30 s. The VHHs were immobilized on a His1K biosensor. The measurement baseline was measured in kinetic buffer for 30 s. The loaded sensors were dipped into 400 nM HER2-Fc (ACROBiosystems, Newark, DE, USA) and GPC3-Fc (ACROBiosystems) to measure a 100-s specific binding at the association step. The dissociation was obtained by dipping the biosensors one more time into the kinetic buffer for 100 s. VHH clones with a binding response of >0.1 were identified as a binder, and sequence analysis was conducted on those clones.

### 2.6. VHH Expression and Purification

The VHH candidates identified by screening were expressed in Corynebacterium, and purified by His tag at the C-terminus of VHHs. The bacterial cells were cultured in CM2G medium at 30 °C overnight. The cells were inoculated in a protein-expression medium and cultured at 25 °C for 72 h. The culture supernatants obtained by centrifugation were filtrated by a 0.22-µm membrane filter and subjected to Ni-NTA HP (FUJIFILM Wako Pure Chemical Corporation, Osaka, Japan) in a spin-column. The resin was washed with the Tris buffer (50 mM Tris-HCl pH7.5, 300 mM NaCl) containing 30 mM Imidazole, and then VHHs were eluted with the Tris buffer containing 500 mM Imidazole.

### 2.7. Binding Affinity Analysis

Binding affinities of VHHs were determined using BLI, Octet^®^ RED384. A solution of 70 μL was added to a 384-well black plateand the measurements were performed as described below. The loading baseline was measured in the kinetic buffer for 30 s. The purified VHHs with C-terminal His tag were immobilized on a His1K biosensor. The measurement baseline was measured in the kinetic buffer for 30 s. The loaded sensors were dipped into two-fold serial dilutions from 100 nM of the HER2-Fc and GPC3-Fc to measure a 120-s specific binding at the association step. The dissociation was obtained by dipping the biosensors into the kinetic buffer for 300 s. The data were analyzed using ForteBio Octet Date Analysis HT 11.1.2.48, and kinetic parameters were determined.

### 2.8. Thermal Stability Analysis

The purified VHHs were analyzed for their thermal stability using differential scanning fluorimetry (DSF) and static light-scattering (SLS) assays. A heating rate of 1 °C/min was monitored from 25 to 95 °C. The SLS assays were measured at 266 nm. Additionally, T_m_ and T_agg_ from the DSF assay were analyzed and calculated using UNCLE Analysis Software (Unchained Labs, Pleasanton, CA, USA).

### 2.9. Cell Culture

SK-BR-3 cells were purchased from the American Type Culture Collection (ATCC, Manassas, VA, USA) and maintained in McCoy’s 5A (Gibco, Grand Island, NE, USA) medium, supplemented with 10% fetal bovine serum (FBS, Sigma–Aldrich, Merck KgaA, Darmstadt, Germany), penicillin (100 U/mL), and streptomycin (0.1 mg/mL) in a humidified 5% CO_2_ incubator. HepG2 cells were purchased from the Japanese Collection of Research Bioresources Cell Bank (JCRB, Osaka, Japan) and maintained in Dulbecco’s Modified Eagle’s Medium (DMEM; Sigma–Aldrich), supplemented with 10% FBS, penicillin (100 U/mL), and streptomycin (0.1 mg/mL) in a humidified 5% CO_2_ incubator.

### 2.10. Flow Cytometry

The purified VHHs were analyzed for cell binding using a flow cytometer. Cells were cultured in dishes at 80% confluence and then harvested with 5 mM EDTA in PBS. Collected cell samples were washed with 1% BSA/PBS, resuspended with the VHH solution in 1% BSA/PBS (1:100 dilution), and incubated at 4 °C for 1 h. After being washed three times with 1% BSA/PBS, the cells were incubated with an anti-His-tag antibody conjugated with Alexa Fluor 488 (1:1000 dilution, Medical & Biological Laboratory, Tokyo, Japan) at 4 °C for 1 h. After incubation with the detection antibody, cells were washed with 1% BSA/PBS, resuspended with 1% BSA/PBS, and subjected to a flow cytometry (FCM) analysis (SH800; Sony, Tokyo, Japan).

### 2.11. Molecular Dynamics Simulations of VHHs

Although our libraries were carefully designed on the basis of the bioinformatics explained above, we needed to check whether the VHHs in the libraries had the expected CDR3 structures. To verify whether the VHHs had the expected CDR3 structural types, we conducted molecular dynamics (MD) simulations of representative sequences from the libraries. The initial structure was created by homology modeling, using the SWISS-MODEL web server [37]. Generally, loop structures such as CDR3 require a very long simulation time due to their slow relaxation, which is difficult to access with conventional MD simulations. Thus, to efficiently sample CDR3 structures, we used the generalized replica-exchange solute-tempering method (gREST) [38], a variant of replica-exchange MD [39]. In the replica-exchange MD, temperatures of whole replicated systems are exchanged to accelerate structural sampling. In the gREST, we could select a local region of the molecule and a part of the potential energy terms for temperature exchange. We found that the CDR3 structure can be efficiently sampled when selecting the CDR3 region and its dihedral angle potential energy terms for the temperature exchange (Higashida and Matsunaga, submitted). Following this procedure, we conducted 100-ns gREST simulations with eight replicas (i.e., 800 ns aggregation time). Finally, we obtained an ensemble of the VHH structure at 1 atm and 300 K, which corresponds to the lowest temperature of the eight replicas. The simulations were conducted with GENESIS software [40] using the CHARMM36 force field [41] and the TIP3P water molecule. Electrostatic interactions were treated using the smoothed particle mesh Ewald (S-PME) [42], and the bonds involving hydrogen atoms were constrained using SHAKE [43] and SETTLE [44] algorithms.

## 3. Results

### 3.1. Classification of CDR3 Structure

To analyze the structural features of camelid VHH, we created structural datasets of VHH and human VH. First, we obtained the structural data of VHH and human VH available in PDB, and then extracted the data with a resolution better than 2.8 Å by X-ray crystallography and excluding the perfect match sequences. As a result, we constructed a dataset of 330 sequences of VHH and a dataset of 926 sequences of human VH.

**Figure 1:**
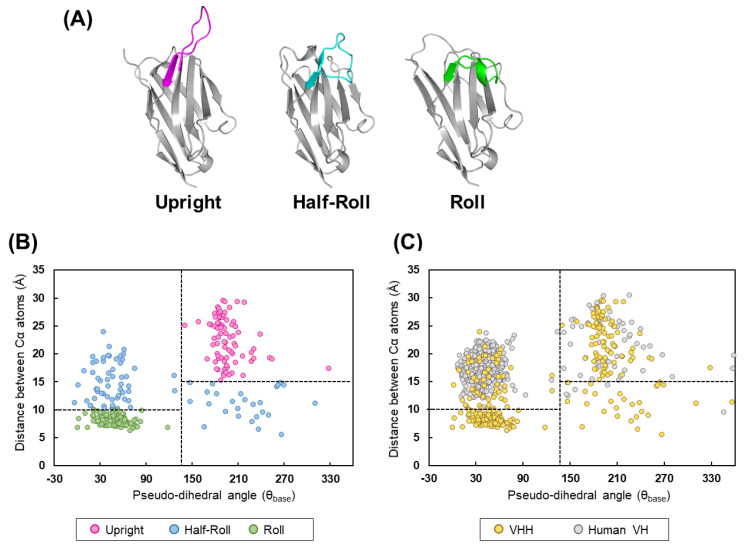
Classification of VHHs by CDR3 loop structures. (**A**) The structure of VHHs is classified into three types based on the conformation of CDR3; *Upright*, *Half-Roll* and *Roll* (PDB ID: 5j1s, 6ru3, and 2p45). (**B**,**C**) The distances between Cα atoms of CDR3 loop and FR2 (distance between H46 residue and (n − 5)th residue with H102 as (n)th residue) and pseudo-dihedral angles of CDR3 C-terminal (dihedral angle consisting of the four Cα atoms of the (n + 1)th, (n)th, (n − 1)th, and (n − 2)th residues, with H102 as the (n)th residue) were calculated from structures and the value was plotted. (**B**) The structures of CDR3 were classified into the three groups according to type (*Upright*, *Roll*, and *Half-roll*) based on the distance and pseudo-dihedral angle. The VHHs belonging in the *Upright* group (pink dots) had a distance of 15Å or more and an angle of 140° to 360°, the VHHs in the *Roll* group (green dots) had a distance of 10Å or more and an angle of less than 140°, and the other VHHs were classified as the *Half-Roll* type (blue dots). (**C**) The CDR3 of human VH (gray dots) from a conventional VH–VL complex and VHH (yellow dots) are shown.

We analyzed the constructed VHH structure dataset and found that the structure of the CDR3 loop can be roughly classified into three groups. Based on the characteristics of the CDR3 structure, each classification was named “*Upright*,” “*Half-Roll*,” and “*Roll*” (Figure 1A). In the *Upright* type, the CDR3 stands upright and the FR2 is exposed to the outside. This *Upright* loop of VHH stab into an epitope of the target antigen, or binds to the target with FR2. The *Roll* type of VHHs has the formation that the CDR3 loop covers the FR2 region. For this formation, large hydrophobic residues (H37, H44, H45, H47) are directly related. *Half-roll* is categorized as the intermediate CDR3 type between *Roll* and *Upright*. Compared with other types of CDR3, this type does not have a clear propensity in its sequence. Next, for those CDR3 loop structure classifications (*Upright*, *Half-Roll*, and *Roll*), the classification was defined by combining the CDR3 classification methods in past studies [32,33]. Figure 1B shows a scatter plot of the pseudo-dihedral angles of CDR3 C-terminal and the distances between the Cα of the CDR3 loop and the closest residue in FR2 for the structures of VHH. The structures with pseudo-dihedral angles more than 140°, distances more than 15 Å, and an elongated-type CDR3 conformation were classified as *Upright* (pink dots). Structures with a pseudo-dihedral angle less than 140°, distances less than 10 Å, and a CDR3 conformation with a curved shape relative to FR2 were classified as *Roll* (green dots). The group of VHHs that do not belong to *Upright* and *Roll* was classified as *Half-Roll* (blue dots) because the CDR3 conformation was intermediate between *Upright* and *Roll*. We determined the regions of CDR3 in human VH and classified the structures by applying the same scheme as we did for VHH. In human VH, there were no *Roll* types and few *Upright* types for CDR3 (Figure 1C). Although most of the structures were *Half-Roll* type, few showed pseudo-dihedral angles more than 140° and less than 15 Å in distance. The presence of a light chain is a steric hindrance and will prevent folding, as with the *Roll*-type structures of CDR3. The above suggests that *Upright* and *Roll* are unique CDR3 structures of VHH that are rarely found in human VH.

**Figure 2:**
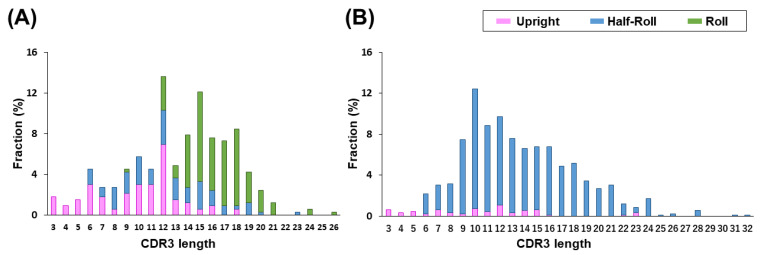

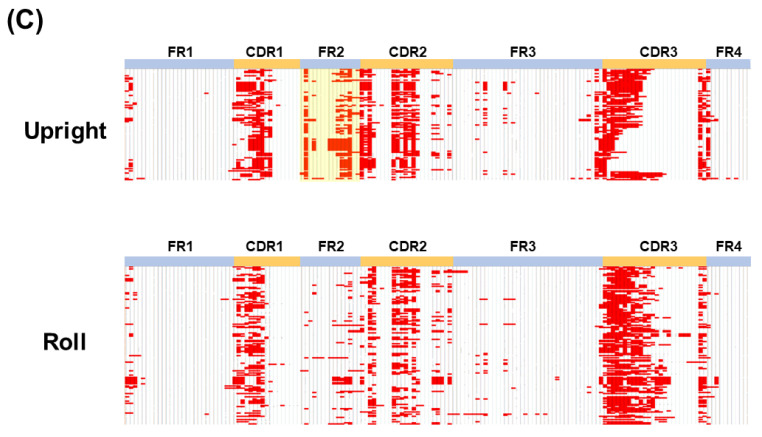
Distribution and structural classification of CDR3 loop lengths of (**A**) VHH and (**B**) Human VH. Many VHHs with CDR3 lengths of 12 amino acids or more belong to *Roll* (green), and VHHs or human VHs with short CDR3 tend to belong to *Upright* (purple). Those belonging to the *Half-Roll* group (blue) are widely distributed. (**C**) Comparison of amino acid residues between *Upright* and *Roll* types involved in the paratope and surroundings. Amino acid residues of VHH close to the antigen are highlighted in red in the crystallography of VHH–antigen complexes listed in PDB. In particular, the FR2 of the *Upright* type (highlighted in yellow) shows that many amino acid residues directly contribute to the paratope formation.

Figure 2A,B shows a histogram of the length of CDR3 in the three structure types of VHHs and human VH from the PDB. In VHH, CDR3s with 12 or more amino acids tend to form a *Roll*-type structure. Alternatively, shorter a CDR3 tends to form an *Upright* conformation in both VHH and human VH. The *Half-Roll* type is widely distributed throughout and independently forms the length of the CDR3. Moreover, CDR3s with 16 or more amino acids tend to have a cysteine residue in the CDR3 that forms a disulfide bond with a cysteine in the CDR1 or CDR2 to stabilize the CDR3 structure. This additional disulfide bond is unwanted in the process development of biologics. Therefore, the maximum length of CDR3 was determined to be 15 amino acids. Furthermore, we found a correlation between CDR2 length and CDR3 structural classification. A total of 87% of the *Roll*-type VHHs in the dataset had 17 amino acid CDR2s, and 62% of the *Upright*-type VHHs had 16 amino acid CDR2s. This suggests that the length of CDR2 affects the structure determination of the CDR3 loop.

Detailed information on the paratope structure was obtained from the X-ray crystallographic analysis of VHHs bound to the antigen (Figure 2C). Initially, we precisely defined CDRs using the Kabat numbering scheme. From the paratope analysis, Kabat numbering of CDR2 (H50–H65) and CDR3 (H95–H102) contained a sufficient region of the paratope. However, since the paratope of the CDR1 region is wider than that defined by the Kabat numbering system, we defined CDR1 (H26–H35) as the combined region of Kabat numbering (H31–H35) and the Chothia-aligned CDR1 (H26–H32). In the *Upright* type, FR2 plays a major role in paratope formation. The FR2 interacts with the L chain in a conventional VL–VH format, but is exposed in the VHH with an *Upright* CDR3 loop. Alternatively, in the *Roll* type, the FR2 and CDR3 are close to each other, stabilize as a *Roll* structure, and the entire CDR3 recognizes the antigen.

### 3.2. Construction of the PharmaLogical Library

**Figure 3:**
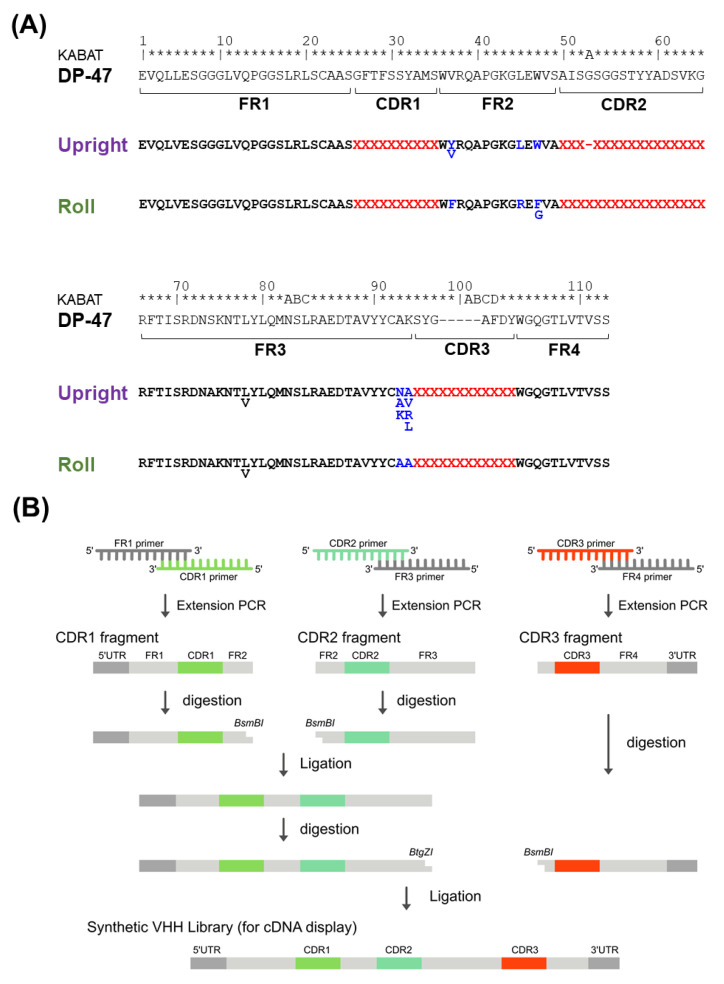
(**A**) The design of the humanized framework regions of the *Upright* and *Roll* sub-libraries and the humanized reference DP-47 are shown. The randomized CDR regions are shown in red. Sequences that differ between the *Upright* library and the *Roll* library are shown in blue. Numbering is according to the Kabat classification scheme. (**B**) The schematic diagram of the library construction process.

We designed a rational artificial library of VHHs based on the analysis of the paratope, residue length, and structural type of CDR3, and, based on a huge number of camelid VHH amino acid sequences accumulated in our laboratory, the frequency of the appearance of all amino acid residues on VHH was analyzed. Four sub-libraries were designed and designated *Upright 6*, *Upright 12*, *Roll 12*, and *Roll 15* according to the structure type and the length of CDR3 loops. First, the humanization framework area of the VHH library was designed for the *Upright* and *Roll* types, respectively. The humanized framework sequence was determined based on the human framework sequence IGHV3-23 * 01 (DP-47) and the humanized sequence of VHH currently on the market (Figure 3A). Because humanization often reduces the binding activity, it is advantageous to have already-humanized VHHs in the library. The CDR3 of the synthetic library was designed to have 6 and 12 amino acids in the *Upright* type sub-library, and 12 and 15 amino acids in the *Roll* type sub-library, based on the analysis of the structural classification and length distribution of CDR3. Furthermore, because some amino acids become modified during the development process, our library was designated to reduce the frequency of appearance of potentially risky amino acids in CDR3. For CDR1 and CDR2, sequences were selected from the alpaca germline and the alpaca sequence data obtained in our laboratory. In this artificial VHH library, CDR1 consists of 150 sequences, and CDR2 consists of 71 sequences of 16 amino acids in the *Upright* type and 69 sequences of 17 amino acids in the *Roll* type. Importantly, the structural properties of camelid VHH were kept in the design of the CDRs.

Based on the library design, four types of sub-libraries, *Upright 6*, *Upright 12*, *Roll 12*, and *Roll 15*, were constructed. Each constructed library had a diversity of more than 10^13^ and was synthesized by adding a UTR sequence for the cDNA display method (Figure 3B). The constructed library was analyzed by next-generation sequencing (NGS) and verified. As a result of NGS analysis, the four sub-libraries had the sequences as designed, and the mixing ratio of the amino acids of CDR3 was almost the same as designed.

### 3.3. Validation of PharmaLogical Library

**Figure 4:**
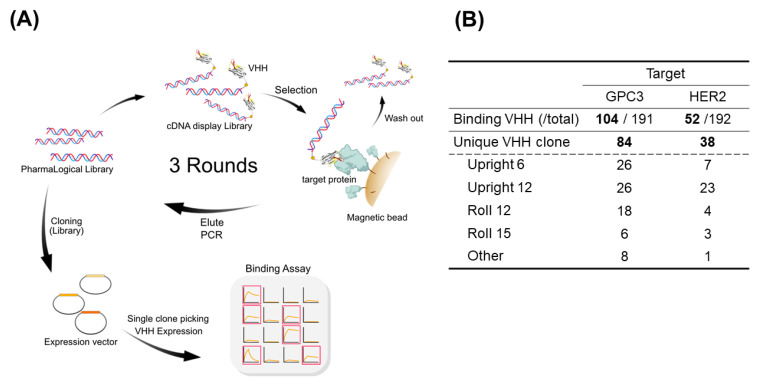
(**A**) Schematic of the screening flow using “*The Month*” platform. The cDNA display selection strategy is used to isolate VHHs that bind to target molecules from the *PharmaLogical* library. The *PharmaLogical* Library, with > 1.0 × 10^13^ diversity, was made into a cDNA display molecule with genotype and phenotype correspondence. Only the cDNA display molecules that bind to the target protein are immobilized on the magnetic bead via the target protein, and the unbound cDNA display molecules were washed away. The DNA of the recovered cDNA display molecule was amplified by PCR, and the PCR product was used as a template in the next cycle. Three-cycle selection was conducted with these series of flows as one cycle. The DNA Library subjected to the selection cycle was cloned into a protein-expression vector, and each clone was expressed. VHHs in the culture supernatant were collected and the binder was identified using Octet384. (**B**) The number of binding VHHs detected as hit clones in the primary binding assay is shown. Unique VHHs are clones detected as hit clones but that do not have duplicate sequences. Unique VHHs were derived from four libraries: *Upright 6*, *Upright 12*, *Roll 12*, and *Roll 15*. VHH clones whose origin could not be determined due to mutations in the screening process were designated other.

The combination of the *PharmaLogical* library with the most current cDNA robust screening method showed robust VHH screening. Various VHHs were quickly obtained from the cDNA display based on our VHH screening platform, “*The Month*” (Figure 4A). A mixture of the four types of sub-libraries from the *PharmaLogical* library was used for screening against human HER2 and human GPC3. In both validation studies, various binders with high affinity were efficiently isolated for each antigen within a month’s time.

Figure 4B shows the outcome of validation studies using the *PharmaLogical* library with our VHH screening platform “*The Month*”. It was confirmed that a wide variety of VHHs was obtained by combining the synthetic VHH library and our VHH screening platform based on the cDNA display method. For GPC3, 104 clones were isolated and, among them, 84 were unique clones. For HER2, 52 clones were obtained and 38 were unique clones. Very interestingly, for HER2, 70% of clones were derived from the *Upright 12* sub-library. The Upright VHHs are most likely to bind to the epitopes, such as crevices and clefts on the antigen, to which conventional antibodies cannot bind. Therefore, the upright VHHs are expected to have new binding characteristics compared to those of conventional antibodies. For GPC3, 50% were from the *Upright 6* and *Upright 12* sub-libraries.

**Figure 5:**
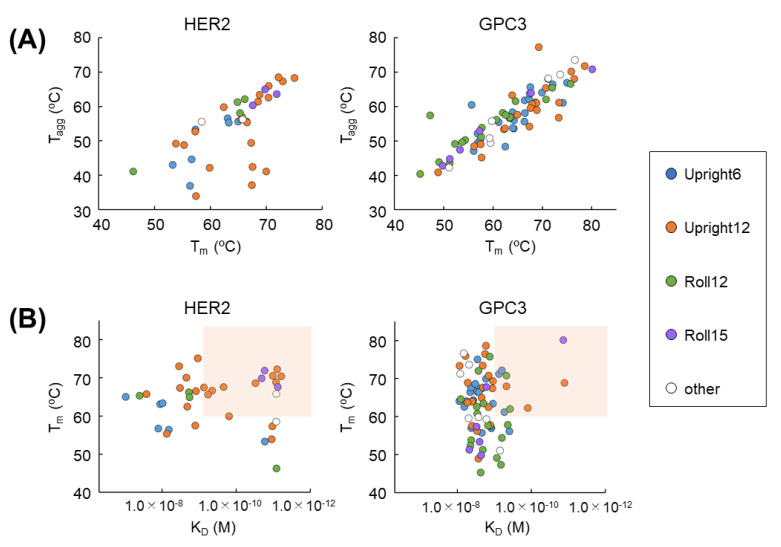
Characteristic of obtained VHHs. (**A**) T_m_–T_agg_ and (**B**) K_D_–T_m_ diagram of unique VHH clones against HER2 and GPC3, shown for the sub-library origin of each VHH clone: *Upright 6* (blue), *Upright 12* (orange), *Roll 12* (green), *Roll 15* (purple), and other (white). The region of high affinity (<1.0 × 10^−9^ M) and high thermal stability (>60 °C) is highlighted in red.

Each unique clone was expressed in *Corynebacterium glutamicum*, which secrete recombinant protein into a culture supernatant. The supernatants were filtered through a 0.22-µm membrane filter, and subjected to analysis of K_D_, T_m_, and T_agg_ by BLI (Octet RED 384, Pall Life Sciences) and the UNCLE system (Uncle Analysis Software, Unchained Labs, Pleasanton, CA, USA), respectively. As shown in Figure 5A, many VHHs obtained from the synthetic VHH library showed high T_m_ and T_agg_ values. Moreover, a T_m_–T_agg_ plot shows a good correlation, especially in GPC3. Binding affinity analysis confirmed that VHHs obtained from the synthetic VHH library have a binding affinity of approximately 10^7^ to 10^−12^ M for each antigen (Figure 5B). A K_D_–T_m_ plot is very useful for selecting candidates for further development (K_D_ < 1.0 × 10^−9^ and T_m_ > 60 °C) (Figure 5B, red-colored box).

**Figure 6:**
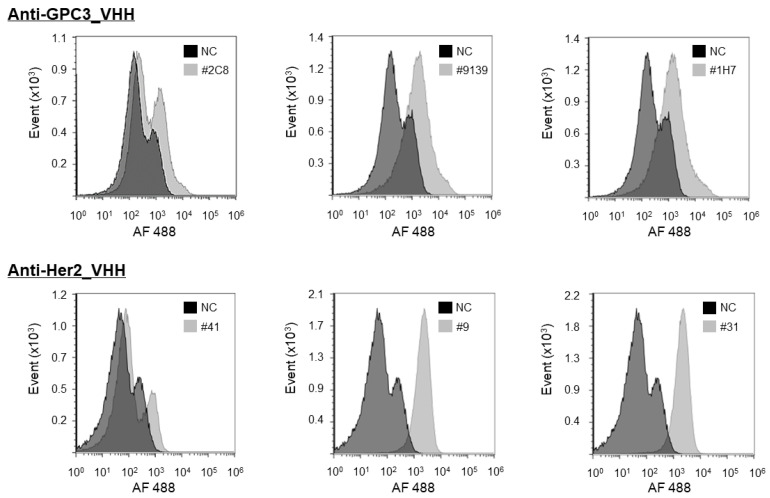
FCM analysis of cell-binding on SK-BR-3 (highly expressed HER2) and HepG2 (highly expressed GPC3). Anti-His-tag mAbs conjugated with Alexa Fluor 488 were used as the secondary antibody, cell binding was detected by the secondary antibody recognizing the C-terminal His tag of VHHs. Negative control (NC) was stained with secondary antibody only.

Flow cytometry (FCM) analysis confirmed a cell-binding activity of VHHs against target antigens. When screening VHHs for peripheral membrane proteins, soluble recombinant proteins were usually used, and thus, sometimes isolated VHHs cannot bind to the antigen on living cells. For clinical applications, therefore, an FCM evaluation needs to select VHHs that bind to target molecules on living cells. Several VHHs isolated by screening were analyzed for cell-binding to SK-BR-3 (highly expressed HER2) and HepG2 (highly expressed GPC3). In this FCM analysis, cell-binding was detected by recognition of the His tag at the C-terminus of VHHs by the Anti-His-tag mAbs conjugated with Alexa Fluor 488. As shown in Figure 6, some VHHs for both GPC3 and HER2 bind to their target liver cancer cell hepG2 and breast cancer cell SK-BR-3, respectively. Therefore, it was confirmed that VHHs obtained from this screening platform using recombinant proteins also showed the binding ability to target molecules on the cell surface.

### 3.4. Confirmation of Reproduction of Paratopes by Molecular Dynamics Simulation

**Figure 7:**
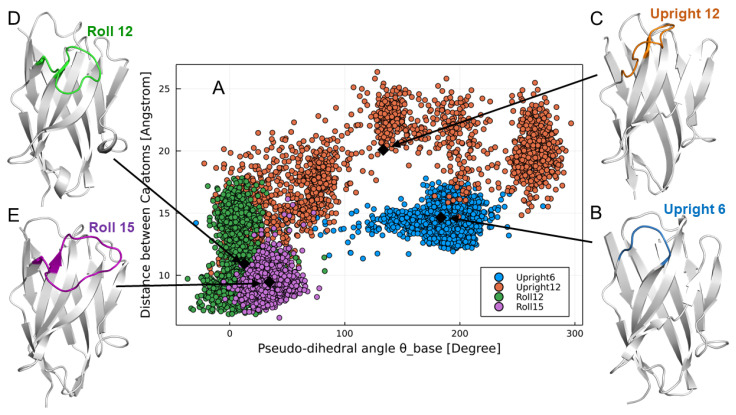
Molecular dynamics simulations of VHHs. (**A**) Simulated VHH structures were mapped onto the distance and the pseudo-dihedral angle space (see Figure 2). *Upright 6* (blue), *Upright 12* (orange), *Roll 12* (green), and *Roll 15* (purple) simulated structures of representative sequences are shown. A representative structure of each VHH from the closest average value in the 2D space is illustrated for *Upright 6* (**B**), *Upright 12* (**C**), *Roll 12* (**D**), and *Roll 15* (**E**).

To verify whether the VHHs have the expected CDR3 structural types, we conducted MD simulations of representative sequences from the sub-libraries. Figure 7 shows the results of gREST simulations of the four representative VHHs from sub-libraries *Upright 6*, *Upright 12*, *Roll 12*, and *Roll 15*, respectively. The obtained structures were projected onto the same distance and pseudo-angle space as the bioinformatics analysis. It was confirmed that the structural ensembles of the VHHs were distributed in the expected *Upright* or *Roll* regions defined in the bioinformatics analysis. Particularly, clones from the *Upright 6*, *Roll 12*, and *Roll 15* are spatially located in typical *Upright* and *Roll* regions. Although a clone from *Upright 12* showed a broad distribution which included the *Half-Roll* region as a metastable state, most stable states correspond to the upright region. In fact, the average value of *Upright 12* (black diamonds) in this 2D space located in the typical upright region was confirmed by the 3D structural view.

## 4. Discussion

There are several approaches to the construction of human VHH libraries. Binders for the COVID-19 spike protein were isolated from a human VHH library, indicating that the human VH could be used in the VHH format [45]. However, it is unclear whether human VH reproduces the structural properties of camelid CDR3 that make a paratope in a single-domain antibody. A comparison between VHH and human VH revealed that the camelid VHH has three types of CDR3 structures, whereas for human VH, the *Half-Roll* type is dominant. This is due to the presence of VL in the conventional antibody and may influence the paratope formation. Since the VHH in our *PharmaLogical* library retains the paratope-forming ability of VHH, it is expected that the VHH will reproduce the binding characteristics of camelid VHH. Therefore, *PharmaLogical* is a logical library that will greatly contribute to antibody drug discovery.

Detailed structural data of the paratope structures were obtained from the X-ray structural analysis of antigen-bound VHH. Compared to computer structure analysis based on the amino acid sequence of CDR, this analyzes the state of binding to the actual antigen, and so more accurate structure data can be obtained. We found that the VHH CDR3 produced by camelids followed three major structural pattern forms. The relationship between CDR2 and CDR3 is particularly important, and there are two types of CDR2 with lengths of 16 or 17 amino acid residues, which defines the possible structural patterns of CDR3. We believe that our artificial library that retains the expected basic structural relationship of VHH is extremely important for reproducing various structures to take advantage of the inherent characteristics of the CDR3 of VHH antibodies.

A synthetic library construction based on universal FRs has been reported [26,27]. However, we found that the *Upright* and *Roll* types interact structurally differently between CDR3 and FR2, and thus, FR2 sequences need to be optimized for each type. That is, a universal FR in the same structure type makes sense, but not for all structure types. Amino acid residues in the FRs generally contribute to direct binding and/or a paratope formation. As shown in Figure 2C, amino acid residues in the FR2, especially in the *Upright* type, contributed to the paratope formation. Therefore, for synthetic libraries, the FR2 should be carefully designed and introduced into the library (Figure 3A).

The usefulness of VHHs has been widely recognized in drug discovery. Not only are the characteristics of VHHs attractive, but also the speed at which drug candidates can be obtained using VHHs, especially in an emergency, is advantageous. Currently, the world is amid a pandemic caused by the coronavirus disease, COVID-19. The time required for developing therapeutic drugs against COVID-19 is a serious issue, and thus, it is expected that VHHs that can be obtained more rapidly from the generation of mutant strains will increase the speed of progress. Wu et al. reported that a yeast surface-display library of synthetic VHHs (>2 × 10^9^) provides neutralizing binders to the spike ectodomain [46]. In addition to designing our library, we built “*The Month*,” a VHH-screening platform that quickly selects development candidates by combining an artificial humanized library with >10^13^ diversity and a cDNA display technology. Thus, our screening platform, named “*The Month*”, is a powerful tool for a rapid response to the outbreak of coronavirus mutants.

As a model case of drug discovery, HER2 and GPC3 were subjected to a VHH drug-discovery process. We used “*The Month*,” our cDNA display screening platform that allows the selection of 50–100 positive clones and analyzes their K_D_, T_m_, and T_agg_. This process was completed within one month, and was, thus, named “*The Month*.” Two-dimensional plotting with some parameters can be created. For example, when plotting T_m_ against T_agg_, a high correlation is obtained in GPC3 (Figure 5A). A correlation also can be seen in HER2. In other words, the selection of a clone population with high T_m_ and T_agg_, which determines protein stability, as a development candidate is confirmed. Additionally, when viewed in terms of K_D_ and T_m_, development candidate VHHs with high affinity and high stability can be selected. Determining the window setting (in this study, K_D_ < 1.0 × 10^−9^ M, T_m_ > 65 °C) quickly narrows down the number of candidates for development. After that, various cell assays can be applied to further obtain candidates to develop. It is useful to quickly select candidates by using “*The Month*” and two-dimensional plot analysis. The purpose of this FACS evaluation study is to evaluate whether VHHs obtained using a soluble antigen can bind firmly to the true target membrane-binding antigen. Therefore, control cells are not always necessary. Of course, it is very important to verify the characteristics of VHH in clinical development. At the next step, therefore, we will investigate the cross-reactivity of VHHs to wide variety of cells. This is an important process that forms the basis of safety assessments in animal and human clinical trials. We will report the results of cellular specificity in the near future.

The most important feature of the *PharmaLogical* library is that it reproduces the paratope formation of camelid VHHs. That is, a selected VHH should reflect the concept of the structural design of the library. To confirm this, the CDR3 structures of selected VHHs were subjected to computer modeling, which is less time-consuming than crystal structure analysis. As expected, the CDR3 structure of the clone derived from the *Roll* sub-libraries clearly showed the *Roll* type structure. Therefore, our *PharmaLogical* library is guaranteed to be composed of the designed structural subtypes. Many VHH libraries have been reported, but our *PharmaLogical* library is the first library to deploy the original structural characteristics for camelid VHHs. Reproducing the original structural properties will lead to more success in the drug-discovery application of VHHs.

Furthermore, Zimmermann et al. also reported a synthetic VHH library [47]. The idea of this paper is similar in that it, as our research does, features a structure-based design. However, they specialize for complex membrane proteins such as transporters and ion channels and focus on epitope structure. On the other hand, we are trying to reproduce the binding properties of camelid antibodies in an artificial humanized library for any kind of targets, focusing on paratope structure. There are structural relationships between epitopes and paratopes, and thus, we expect that this interesting structural relationship could be useful in a structure-based drug design.

## 5. Conclusions

The library we logically designed based on structural analysis makes it possible to secure VHHs that reproduce the attractive paratope of the camelid VHH. Currently, although we have completed the construction of the *Half-Roll* type sub-libraries, we are in the process of undergoing validation. Once this is complete, we will be able to provide an artificial library that covers about 95% of the original camelid VHHs. *PharmaLogical* is an in vitro antibody production system that completely mimics the VHH production of an in vivo system. Furthermore, in the future, it may be useful to select the appropriate sub-library according to the antigen.

## Data Availability

The data presented in this study are available within this article.
